# Guard Cell Chloroplasts Are Essential for Blue Light-Dependent Stomatal Opening in Arabidopsis

**DOI:** 10.1371/journal.pone.0108374

**Published:** 2014-09-24

**Authors:** Noriyuki Suetsugu, Tsuneaki Takami, Yuuta Ebisu, Harutaka Watanabe, Chihoko Iiboshi, Michio Doi, Ken-ichiro Shimazaki

**Affiliations:** 1 Department of Biology, Faculty of Sciences, Kyushu University, Fukuoka, Japan; 2 Graduate School of System Life Sciences, Kyushu University, Fukuoka, Japan; 3 Faculty of Art and Science, Kyushu University, Fukuoka, Japan; The University of Tokyo, Japan

## Abstract

Blue light (BL) induces stomatal opening through the activation of H^+^-ATPases with subsequent ion accumulation in guard cells. In most plant species, red light (RL) enhances BL-dependent stomatal opening. This RL effect is attributable to the chloroplasts of guard cell, the only cells in the epidermis possessing this organelle. To clarify the role of chloroplasts in stomatal regulation, we investigated the effects of RL on BL-dependent stomatal opening in isolated epidermis, guard cell protoplasts, and intact leaves of *Arabidopsis thaliana*. In isolated epidermal tissues and intact leaves, weak BL superimposed on RL enhanced stomatal opening while BL alone was less effective. In guard cell protoplasts, RL enhanced BL-dependent H^+^-pumping and DCMU, a photosynthetic electron transport inhibitor, eliminated this effect. RL enhanced phosphorylation levels of the H^+^-ATPase in response to BL, but this RL effect was not suppressed by DCMU. Furthermore, DCMU inhibited both RL-induced and BL-dependent stomatal opening in intact leaves. The photosynthetic rate in leaves correlated positively with BL-dependent stomatal opening in the presence of DCMU. We conclude that guard cell chloroplasts provide ATP and/or reducing equivalents that fuel BL-dependent stomatal opening, and that they indirectly monitor photosynthetic CO_2_ fixation in mesophyll chloroplasts by absorbing PAR in the epidermis.

## Introduction

Light-induced stomatal opening facilitates CO_2_ uptake for photosynthetic CO_2_ fixation and drives the transpirational water stream [1]–[4]. A stoma is comprised of a pair of specialized epidermal cells, so-called guard cells. Stomatal opening is mediated by the sophisticated regulation of ion transport and solute biosynthesis in guard cells [2]–[5]. The accumulation of K^+^ salts, sugars, and malate is brought about by the hyperpolarization of the membrane potential by electrogenic proton pumps (H^+-^ATPases) and metabolic activities in guard cells [1], [2]. Blue light (BL) is a signal inducing stomatal opening caused by the swelling of guard cells [1]. The plasma membrane H^+^-ATPase is activated by phosphorylation of the C-terminal penultimate threonine residue leading to the binding of 14-3-3 proteins, and drives BL-dependent stomatal opening [6]–[8]. Recent molecular genetic analyses in *Arabidopsis thaliana* identified signal transduction components leading to H^+^-ATPase activation and stomatal opening. The plasma membrane-associated receptor kinase phototropins (phot), phot1 and phot2, act as BL-specific photoreceptors [9], and are activated by BL through autophosphorylation [10], [11]. The activated phots phosphorylate the Ser/Thr protein kinase BLUE LIGHT SIGNALING1 (BLUS1) in a BL-dependent manner [12]. The activated BLUS1 finally induces H^+^-ATPase activation via protein phosphatase 1 family proteins (PP1s) [13], although several components have yet to be identified in this pathway. We note that BL inhibits S-type anion channels in guard cells, thereby stimulates stomatal opening in a phototropin-dependent manner [14].

In the leaf epidermis of higher plants except for the orchid genus *Paphiopedilum* [15], guard cells contain photosynthetically active chloroplasts in general [16] which have been suggested to play an important role in stomatal opening [17]. Guard cell chloroplasts are capable of ATP production through cyclic and noncyclic photophosphorylation [18], [19] and generate the reducing equivalents (NADPH) through linear electron transport [20]. However, activities of Calvin cycle enzymes relative to the amounts of chlorophyll present are quite different between mesophyll and guard cell chloroplasts. In guard cell chloroplasts, ribulose-1,5-bisphosphate carboxylase/oxygenase and fructose-1,6-bisphosphatase activities show lower activities, but 3-phosphoglycerate kinase (PGAK), NADP-glyceraldehyde-3-phosphate dehydrogenase (NADP-GAPD), and triose phosphate isomerase (TPI) exhibit much higher activities, compared to mesophyll chloroplasts [20]. The results suggest that ATP and reducing equivalents in the chloroplasts are not fully consumed in CO_2_ fixation, and suggests the transfer of excessive ATP and reducing equivalents to the cytosol via the phosphoglycerate/dihydroxyacetone phosphate shuttle in guard cells [1], [20]. However, the exact role of guard cell chloroplasts in stomatal opening remains unclear [1], [21], [22].

Continuous and high intensities of red light (RL) induce stomatal opening in leaves. The photosynthetic electron transport inhibitor 3-(3,4-dichlorophenyl)-1,1-dimethylurea (DCMU) inhibits this response [23]–[28], indicating that RL-induced stomatal opening is photosynthesis-dependent. However, in several species the contribution of guard cell chloroplasts to stomatal opening is marginal, and RL is not efficient in inducing stomatal opening in the epidermal tissues [28]–[32].

By contrast, there is evidence that guard cell chloroplasts are essential for BL-dependent stomatal opening, and that the chloroplasts synergistically function with BL-specific systems in isolated epidermis and intact leaves. For example, RL induces partial stomatal opening but weak BL superimposed on RL rapidly induces large stomatal apertures [1], [33]–[37]. In the presence of weak BL that does not induce stomatal opening by itself, RL greatly enhances the responses in intact leaves of several plant species [33]–[37]. The decrease of intercellular CO_2_ concentration (*Ci*) by mesophyll photosynthesis under RL enhances the BL-induced stomatal opening [22], [23], [33], [36], [37], [38]. This enhancement by RL is found even in CO_2_-free air [33], implying the existence of some mesophyll factors [28]–[32] and/or the contribution of guard cell chloroplasts [39]. In agreement with the latter notion, the enhancement of BL-induced stomatal opening by RL was found in epidermal peels of *V. faba* and *C. communis* [24]. Furthermore, BL stimulated the formation of malate, which serves as the negative counter ion for potassium ions in guard cells; this malate formation was synergistically enhanced by RL in epidermal peels of *V. faba* [40].

In this study, we investigated the influence of RL on stomatal BL responses and the effects of DCMU on these responses of *A. thaliana*. Our results indicate that the photosynthetic electron transport in guard cell chloroplasts is essential for BL-dependent stomatal opening.

## Materials and Methods

### Plant Materials

Arabidopsis wild type (Columbia *gl1*) and the *phot1phot2* mutant [9] were used. Seeds were sown on soil∶vermiculite (1∶1) and cultured for about 4 weeks under a 14/10 h light/dark cycle.

### Measurement of stomatal aperture in isolated epidermis

Preparation of epidermal tissues from rosette leaves in 4-week-old *A. thaliana* and measurement of stomatal aperture were performed as previously described [10]. Epidermal peels were floated on basal reaction mixture (5 mM mesbistrispropane [pH 6.5], 50 mM KCl, 0.1 mM CaCl_2_) and kept in darkness or under blue and/or red light at various fluence rates for 2 h. For DCMU experiments, 10 µM DCMU (0.05% [v/v] ethanol) or 0.05% [v/v] ethanol alone (as a control) were included in the basal reaction mixture.

### Isolation of guard cell protoplasts

Guard cell protoplasts were isolated enzymatically from rosette leaves as described previously [41]. The protoplasts were kept in 0.4 M mannitol solution supplemented with 1 mM CaCl_2_ on ice in darkness before use.

### Measurement of light-induced H^+^-pumping

BL-induced H^+^-pumping (medium acidification) and RL-induced medium pH changes (medium alkalization and subsequent acidification) were measured with a glass pH electrode as described previously [41]. Guard cell protoplasts corresponding to 50 µg proteins were incubated in pumping buffer containing 0.125 mM MES-NaOH (pH 6.0), 1 mM CaCl_2_, 0.4 M mannitol, 10 mM KCl, and 0.01% DMSO. DCMU was added at the final concentration of 10 µM. The H^+^-ATPase activity was assessed by analyzing fusicoccin-induced H^+^-pumping after the measurement of light-induced medium pH changes.

### Immunoblot and protein blot analyses

Guard cell protoplasts in pumping buffer were dark-adapted and illuminated with 5 µmol m^−2^ s^−1^ BL, or with 55 µmol m^−2^ s^−1^ RL for 2 h followed by 5 µmol m^−2^ s^−1^ BL on a background of 55 µmol m^−2^ s^−1^ RL. Samples for blotting were collected at 0, 3, 5, and 10 min after BL irradiation. Preparation of protein samples and blotting analyses were performed as previously described [6].

### Measurement of stomatal conductance

Stomatal opening in intact leaves was measured by the gas exchange method using LI-6400 open-flow systems (Li-Cor Inc., Lincoln, NE) as described previously [42]. Lighting and experimental conditions were the same as in a previous study [42] (constant CO_2_ concentration of 350 µl l^−1^; 24°C leaf temperature; 55–60% relative humidity. Plants were placed in a 14 ml Falcon tube with distilled water and kept in the dark overnight. Measurements were conducted between 8 a.m. and 5 p.m.

## Results

### Red light enhances blue light-dependent stomatal opening via photosynthesis in isolated epidermal tissues

**Figure 1:**
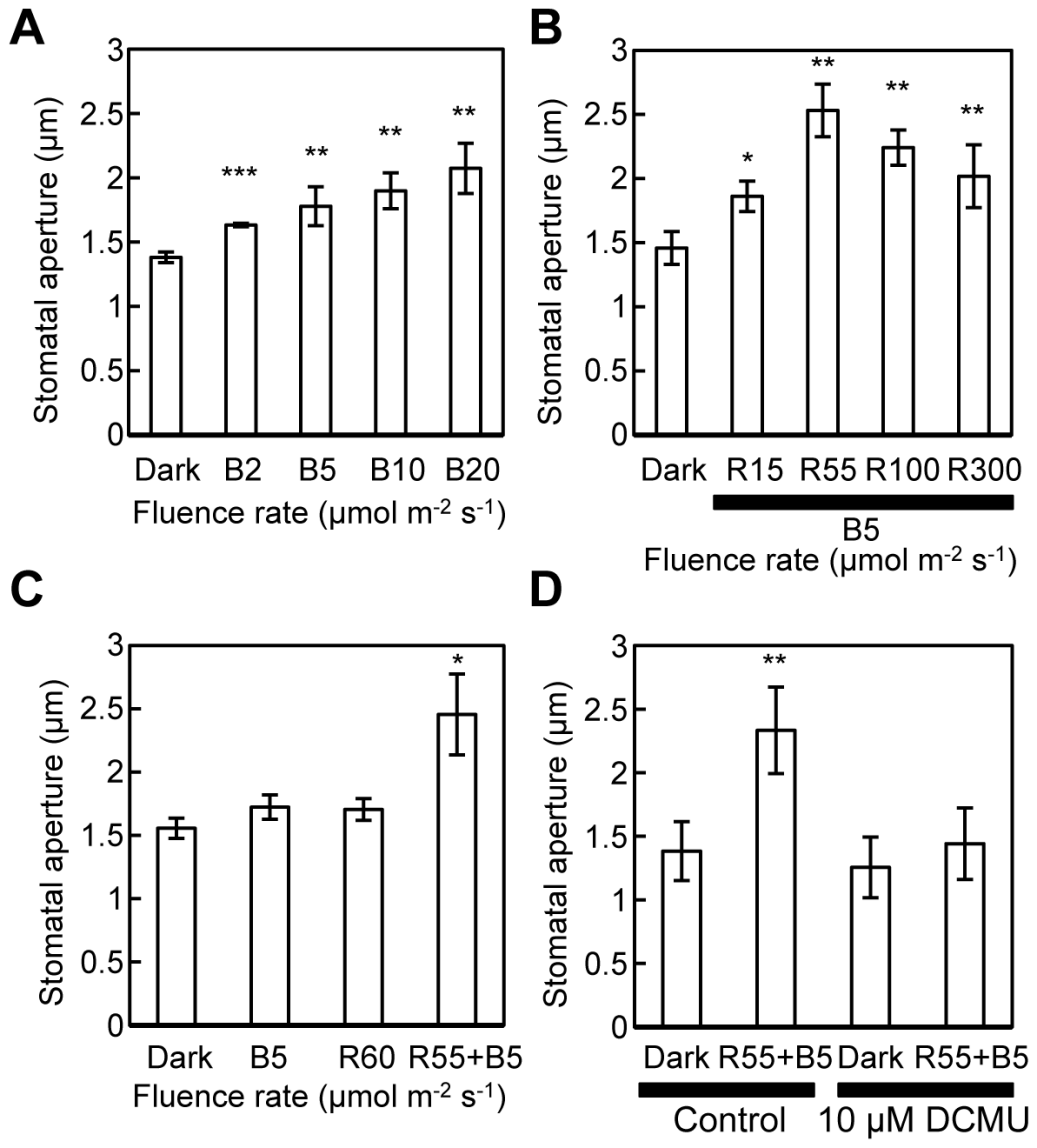
BL-dependent stomatal opening enhanced by RL-activated guard cell photosynthesis. A–D, BL-induced stomatal opening in isolated epidermal tissues of Arabidopsis. A, Stomatal aperture after 2 h incubation in darkness (Dark) or under BL at various fluence rates (B2, B5, B10, and B20 represent 2, 5, 10, and 20 µmol m^−2^ s^−1^ of BL, respectively). B, Stomatal aperture after 2 h incubation in darkness or under BL (B5) superimposed on RL at various fluence rates (R15, R55, R100, and R300 represent 15, 55, 100, and 300 µmol m^−2^ s^−1^ of RL, respectively). C, Stomatal aperture after 2 h incubation in darkness or under 5 µmol m^−2^ s^−1^ BL (B5), 60 µmol m^−2^ s^−1^ RL (R60), or 5 µmol m^−2^ s^−1^ BL plus 55 µmol m^−2^ s^−1^ RL (R55+B5). D, Stomatal aperture after 2 h incubation in darkness or under simultaneous irradiation with BL and RL (R55+B5), with or without DCMU. Data shown are means ± SE of four (A) or three (B–D) independent experiments. For each light condition in an experiment, the apertures of 45 stomata were determined. Asterisks indicate the statistically significant differences compared to Dark control (without DCMU in D), assessed by Student's *t*-tests (*: 0.05<*P*<0.1, **: 0.01<*P*<0.05, ***: *P*<0.01).

To minimize the effect of mesophyll chloroplasts (i.e., the decrease of *Ci*) and explore the role of guard cell chloroplasts, effects of RL on BL-dependent stomatal opening were investigated in isolated epidermal tissues. In our previous study, BL at 5 µmol m^−2^ s^−1^ was sufficient to elicit BL-dependent stomatal opening under 50 µmol m^−2^ s^−1^ RL [9]. In the absence of background RL, stomata subtly opened in response to 5 µmol m^−2^ s^−1^ BL, and stomatal aperture became larger as the fluence rate of BL was increased (Figure 1A). Stomatal aperture was approximately 2 µm under BL at 20 µmol m^−2^ s^−1^ (Figure 1A). In the presence of 5 µmol m^−2^ s^−1^ BL, superimposed RL strongly enhanced stomatal opening, with 55 µmol m^−2^ s^−1^ RL causing the maximum effect (Figure 1B). The stronger intensity of RL than 55 µmol m^−2^ s^−1^ appeared to suppress stomatal opening although the reason for this response was not elucidated at present time. BL at 5 µmol m^−2^ s^−1^ or RL at 60 µmol m^−2^ s^−1^ alone induced only slight stomatal opening, but the simultaneous application induced large apertures (Figure 1C), indicating that BL and RL enhanced stomatal opening synergistically in the isolated epidermis. The enhancement of BL-dependent stomatal opening by RL was eliminated by treatment with 10 µM DCMU (Figure 1D), suggesting that photosynthetic electron transport in guard cell chloroplasts plays an essential role in the enhancement of BL-dependent stomatal opening.

### Red light enhances blue light-dependent H^+^-pumping via photosynthesis in guard cell protoplasts

**Figure 2:**
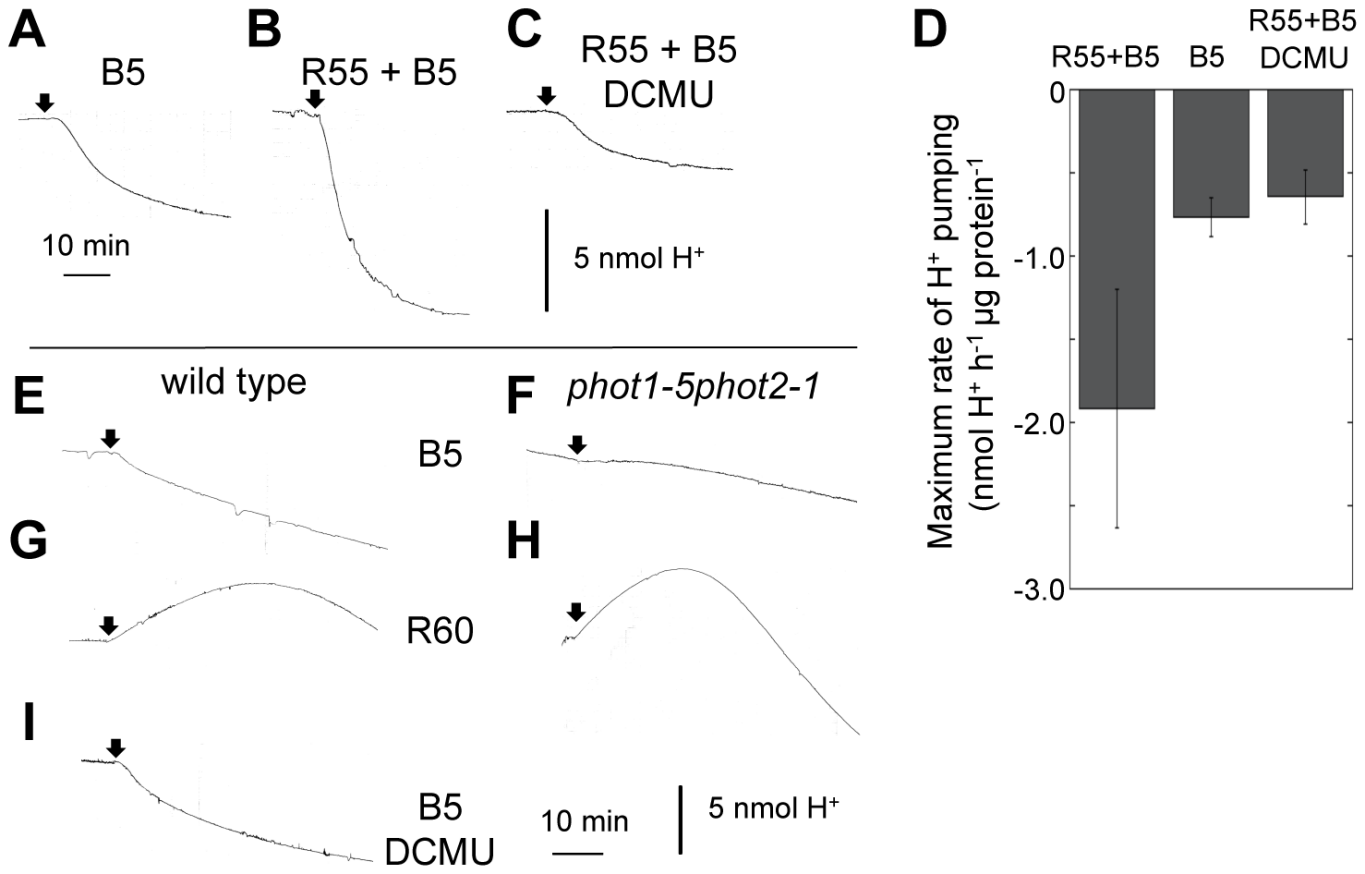
BL-dependent H^+^-pumping enhanced by RL-activated guard cell photosynthesis. A–C. H^+^-pumping induced by the irradiation with BL with (B and C, [R55+B5]) or without RL irradiation (A, [B5]) in wild-type guard cell protoplasts. Guard cell protoplasts (50 µg protein) were irradiated with 5 µmol m^−2^ s^−1^ BL with or without 2 to 3 h pre-irradiation and background irradiation with 55 µmol m^−2^ s^−1^ RL. DCMU (dissolved in DMSO) was administered to the protoplasts before pre-irradiation with RL at a final concentration of 10 µM (0.01% DMSO) (C). D, Maximum rates of H^+^-pumping by guard cell protoplasts under different light conditions as in A to C. Data represent means ± SD of three independent experiments. E–I. pH changes induced by irradiation with BL or RL in wild-type (E, G, and I) and *phot1phot2* (F and H) guard cell protoplasts. Guard cell protoplasts (50 µg protein) were dark-adapted for 1 h before 5 µmol m^−2^ s^−1^ BL (E, F, and I [B5]) or 60 µmol m^−2^ s^−1^ RL (G and H [R60]) was applied where indicated by arrows. DCMU was administered before dark-adaptation at 10 µM (I).

It is possible that guard cell chloroplasts provide the driving force for stomatal opening by producing ATP or reducing equivalents to fuel H^+^-pumping in response to RL [39]. To demonstrate the role of guard cell chloroplasts in BL-dependent H^+^-pumping, extracellular acidification by guard cell protoplasts was measured in the presence or absence of RL. Guard cell protoplasts were irradiated with RL for 2–3 h until the pH had reached a steady state, and then weak BL was applied (Figure 2, A to C). Irradiation of isolated epidermis with BL at 5 µmol m^−2^ s^−1^ had been insufficient to open stomata (Figure 1), and weak BL alone after 2 h in darkness induced a slight acidification (Figure 2A [B5]). Irradiation of guard cell protoplasts with RL at 55 µmol m^−2^ s^−1^ enhanced BL-dependent H^+^-pumping (Figure 2B), and DCMU eliminated the enhancement (Figure 2C). With RL, the maximum rate of H^+^-pumping was more than twice as high as that of guard cell protoplasts with BL alone (Figure 2D).

Contrary to wild-type guard cell protoplasts (Figure 2E), no pH changes occurred in *phot1phot2* protoplasts in response to weak BL at 5 µmol m^−2^ s^−1^ (Figure 2F). Alkalization of the medium was transiently induced by irradiation of guard cell protoplasts from both wild type and *phot1phot2* with RL at 60 µmol m^−2^ s^−1^ (Figure 2G and H [R60]) as a consequence of photosynthetic CO_2_ fixation [43]. The results suggest that weak BL activated phot-mediated signaling but did not activate photosynthesis in guard cells to a detectable level. Furthermore, DCMU did not affect BL-dependent acidification in wild-type protoplasts (Figure 2I), indicating that DCMU had no inhibitory effect on the phot signaling pathway. These results substantiated the synergistic effect between weak BL and RL shown above (Figure 2, A to D). Taken together, our findings indicated that RL enhances BL-dependent H^+^-pumping via the photosynthetic electron transport in guard cell chloroplasts.

### RL effects on the phosphorylation of H^+^-ATPase

**Figure 3:**
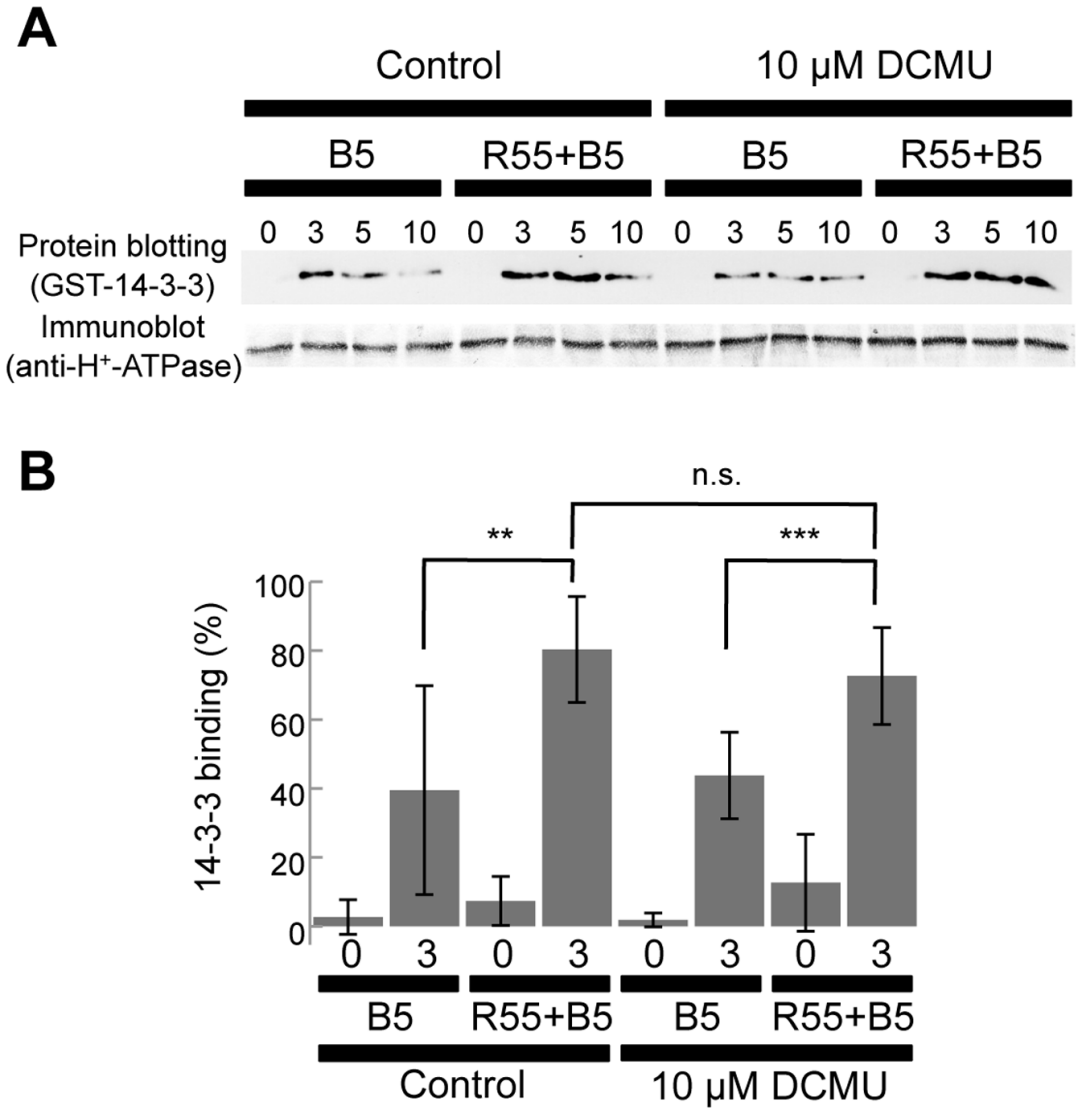
BL-induced phosphorylation of H^+^-ATPase in wild-type guard cell protoplasts. Guard cell protoplasts (60 µg protein) were irradiated with 5 µmol m^−2^ s^−1^ BL with or without 2 h pre-irradiation and background irradiation with 55 µmol m^−2^ s^−1^ RL (R55+B5 and B5, respectively). DCMU (dissolved in DMSO) was administered before pre-irradiation with RL at 10 µM (0.01% DMSO). In samples collected at 0, 3, 5, and 10 min after BL exposure, the phosphorylation status of the H^+^-ATPase was determined by protein blotting with a 14-3-3 protein. Each lane contained 5 or 7 µg guard cell proteins for protein blotting (upper panels) and immunoblotting (lower panels). A, Representative example of similar results obtained in five independent experiments. B, Quantification of the binding of 14-3-3 proteins to the H^+^-ATPase. The 14-3-3 binding before or 3 min after BL exposure was determined. Values presented are means of five independent experiments with SDs. Asterisks indicate the statistically significant differences, assessed by Student's *t*-tests (**: 0.01<*P*<0.05, ***: *P*<0.01). n.s. indicates the statistically insignificant difference (*P*>0.43).

BL-dependent H^+^ efflux is brought about by the activated H^+^-ATPase, and the activation is caused by phosphorylation of the C-terminal domain leading to 14-3-3 binding activity [6]. We investigated whether the effect of RL is correlated with the phosphorylation of the H^+^-ATPase by protein blot analyses with 14-3-3 in guard cells (Figure 3). In guard cell protoplasts that had been dark-adapted or RL-irradiated (55 µmol m^−2^ s^−1^) for 2 h, phosphorylation levels of H^+^-ATPase were low. When guard cell protoplasts were irradiated with weak BL, the phosphorylation levels were increased and stable during the irradiation period (Figure 3A and B). When RL at 55 µmol m^−2^ s^−1^ was applied during the BL irradiation, higher phosphorylation levels of H^+^-ATPase were found although the levels varied between experiments (Figure 3A and B). However, the increase in the H^+^-ATPase phosphorylation by RL was not affected by DCMU (Figure 3A and B). The abundance of H^+^-ATPase was not changed under any conditions (Figure 3A). These results indicated that the H^+^-ATPase phosphorylation level is not directly related to the enhancement of BL-dependent H^+^-pumping by RL (see Discussion).

### Red light enhances blue light-dependent stomatal opening in intact leaves

**Figure 4:**
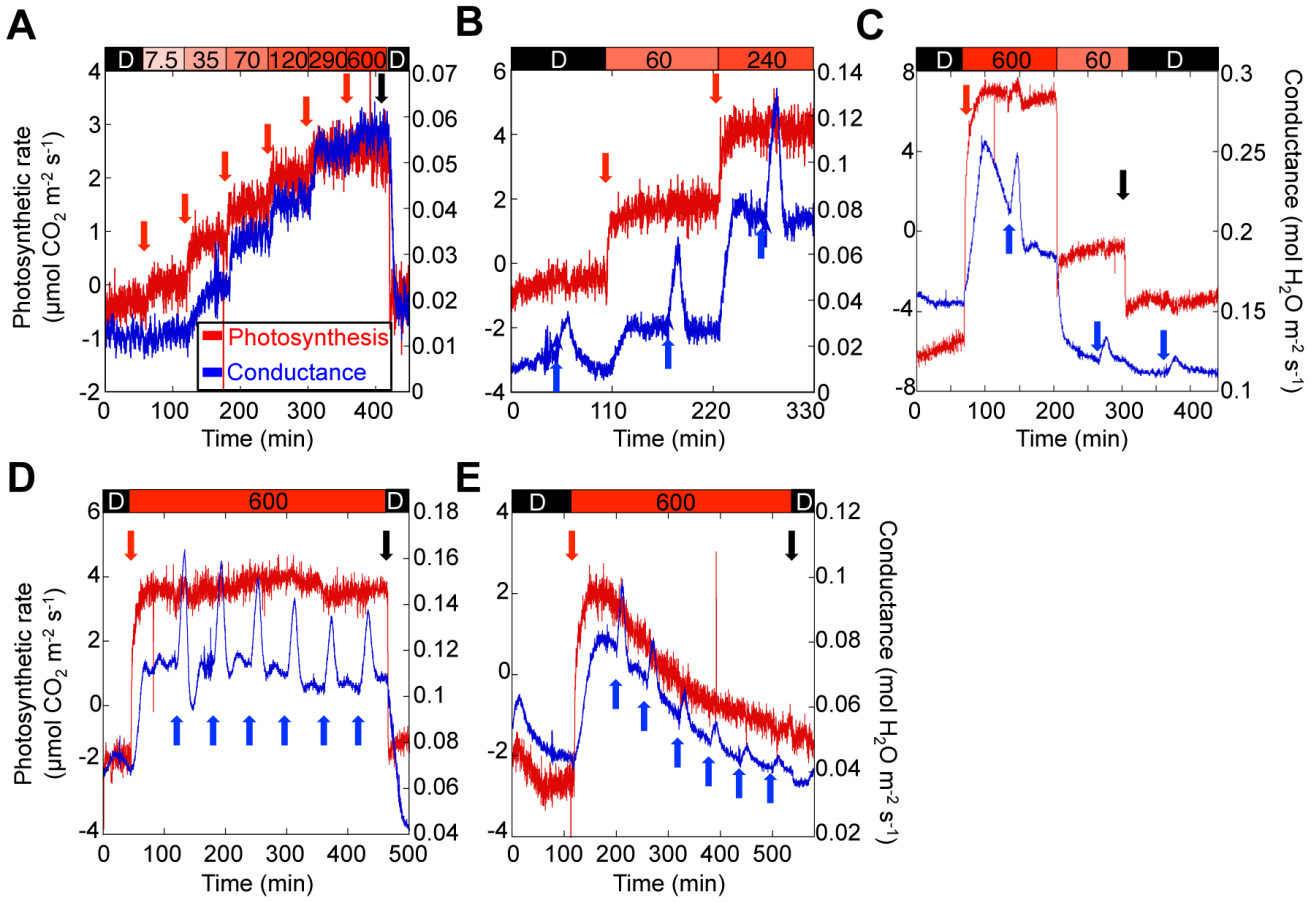
Light-induced increases of photosynthetic rate and stomatal conductance in intact leaves of Arabidopsis. A, Fluence rate-dependency of photosynthetic rate and stomatal conductance in response to RL. B and C, Fluence rate-dependency of BL-induced changes in stomatal conductance in response to RL. D and E, Repeated BL-induced changes in stomatal conductance in a control leaf (D) and a DCMU-treated leaf (E). Photosynthetic rate and stomatal conductance are indicated by red and blue traces, respectively. The timing of fluence rate increases (red and blue arrows) and light-off (black arrows) is indicated next to the corresponding track. The RL irradiation scheme is shown on top of each graph. The irradiation with BL at 5 µmol m^−2^ s^−1^ lasted for 10 min in all cases (light-off markers were omitted for clarity).

Our above results indicated that photosynthetic electron transport activity in guard cells is essential for blue light-dependent stomatal opening in isolated epidermis. We next examined whether RL enhances BL-dependent stomatal opening in intact leaves. First, we determined stomatal conductance in response to RL in leaves using a gas exchange method [42]. Stomatal conductance and photosynthetic CO_2_ fixation increased with increasing RL fluence rate, and both parameters reached a maximum around 600 µmol m^−2^ s^−1^ RL (Figure 4A). Irradiation with 240 µmol m^−2^ s^−1^ RL enhanced BL-dependent stomatal opening more strongly than irradiation with 60 µmol m^−2^ s^−1^ RL (Figure 4B). When the intensity of RL was decreased, the magnitude of the stomatal BL response decreased (Figure 4C). In the absence of RL, BL sometimes failed to induce stomatal opening, depending on the growth conditions of the plants as reported previously [1]. These results indicated that RL is necessary for BL-dependent stomatal opening in intact leaves, and that the photosynthetic rate in leaves correlates positively with BL-dependent stomatal opening.

We then investigated the influence of DCMU on light-dependent stomatal opening and photosynthetic CO_2_ fixation in intact leaves. As a control, we first determined the effects of 0.05% ethanol, the solvent in which DCMU was applied. In the control, BL (5 µmol m^−2^ s^−1^) superimposed on RL (600 µmol m^−2^ s^−1^) for 5 min rapidly enhanced stomatal conductance, and the conductance decreased after turning off the blue light (Figure 4D). The response could be induced repeatedly by brief BL exposure with a slight decrease of BL-dependent stomatal opening over time; importantly, photosynthetic CO_2_ fixation remained unaffected (Figure 4D). The rate of stomatal opening induced by BL was three to four-fold faster than that induced by RL. When 50 µM DCMU in 0.05% ethanol were applied to the leaf through the petiole, both RL-induced stomatal opening and photosynthetic CO_2_ fixation were inhibited increasingly with time, and BL-dependent stomatal opening was similarly inhibited (Figure 4E). This indicated that photosynthetic CO_2_ fixation and RL-induced as well as BL-dependent stomatal opening were affected by DCMU in a closely similar fashion.

## Discussion

Blue light initiates stomatal opening by activating the plasma membrane H^+^-ATPase, and ATP must be continuously provided to sustain H^+^-pumping. Mitochondria and chloroplasts are candidate ATP sources [39], [43]. Guard cells in most plant species possess photosynthetically active chloroplasts [1], [3], [19], [21], [25]. Although a role of guard cell chloroplasts in stomatal opening was implicated in several studies [17], [20], [24], [25], even strong RL was sometimes inefficient to induce stomatal opening in isolated epidermal tissues [10], [12]. To clarify the role of guard cell chloroplasts in the responses, we investigated the effect of RL on BL-dependent stomatal opening in intact leaves, isolated epidermis, and guard cell protoplasts from *A. thaliana*. In isolated epidermal tissues, weak BL did not induce stomatal opening but RL synergistically enhanced the opening with weak BL; this RL effect was eliminated by DCMU (Figure 1). Thus, we conclude that the enhancement of BL-dependent stomatal opening by RL required photosynthetic electron transport in guard cell chloroplasts.

Activation of the plasma membrane H^+^-ATPase in guard cells is a key step for BL-dependent stomatal opening. It results in membrane hyperpolarization which drives K^+^ uptake through voltage-gated K^+^ channels [1], [2], [6], [44]. There is circumstantial evidence from analyses of light-dependent pH changes in the suspending medium of the protoplasts which suggests that RL enhances BL-dependent H^+^-pumping in guard cell protoplasts [45]–[47]. However, the effect of DCMU on the enhancement of BL responses by RL (or white light-induced H^+^-pumping) was controversial; no effects [45], slight suppression (approximately 12%) [47], and substantial (approximately 40–60%) suppression [46]. In these works, pre-illumination of guard cell protoplasts with RL for 30 min or less was performed before BL application, and RL also caused medium alkalization due to CO_2_ fixation [43]. Therefore, the light-induced pH changes determined in these studies might have been the combined effects of H^+^-pumping and the removal of carbonic acid [45]–[47]. To avoid this potential pitfall, we pre-irradiated guard cell protoplasts with RL until the pH had reached a steady state after 2–3 h. As a consequence, we clearly showed that weak BL on an RL background induces strong H^+^-pumping while the same fluence rate of BL alone causes much weaker H^+^-pumping (Figure 2), and that the responses coincided with the behavior of the stomata (Figure 1). Furthermore, DCMU eliminated the RL effect on BL-induced H^+^-pumping and stomatal opening. We concluded that the photosynthetic electron transport driven by RL enhanced BL-dependent H^+^-pumping in guard cells and resulted in a large stomatal apertures.

BL induces the phosphorylation of the H^+^-ATPase necessary for BL-dependent H^+^-pumping [6], and that the phosphorylation levels correlate with the magnitude of H^+^-pumping [6], [7], [12], [13], [41], [48], [49]. We found that RL increased the phosphorylation levels of H^+^-ATPase in the presence of BL (Figure 3) in accordance with increased H^+^-pumping (Figure 2). However, the enhancement of H^+^-ATPase phosphorylation brought about by RL was not significantly inhibited by DCMU (Figure 3). Thus, it is plausible that enhanced phosphorylation is necessary but not sufficient for BL-dependent H^+^-pumping. Discrepancies between the phosphorylation status of the H^+^-ATPase and the magnitude of H^+^-pumping were also found when stomatal opening was elicited by the fungal toxin fusicoccin [39]. Furthermore, since we indirectly determined the phosphorylation levels of H^+^-ATPase by binding amount of 14-3-3 protein, other phosphorylation sites might be involved in the regulation of H^+^-ATPase [7].

The reducing equivalents produced by photosynthetic electron transport in guard cell chloroplasts [20], [50] probably are required for stomatal opening. The reducing equivalents might be utilized for malate synthesis via reduction of oxaloacetate that is produced by the degradation of starch stored in guard cell chloroplasts. In accord with this idea, the malate contents of guard cells [40], [51] increased with stomatal opening in response to both BL and RL. RL synergistically enhanced malate formation in the presence of BL [40]. Alternatively, the reducing equivalents act as redox signaling factor to influence the ion transport in the plasma membrane [22]. Detailed analyses of metabolite changes in guard cells in response to BL alone or combined BL and RL will further unravel the roles of guard cell chloroplasts in these processes.

In conclusion, we have demonstrated that photosynthetic electron transport in guard cell chloroplasts has a crucial function in BL-dependent stomatal opening. The stomatal aperture in intact leaves becomes larger when the intensity of RL is increased in the presence of BL (Fig. 4). Under sunlight, coordinated activation of photosynthesis in mesophyll and guard cells is necessary for efficient photosynthetic CO_2_ fixation, because mesophyll chloroplasts require CO_2_ supplied through stomata that are regulated by guard cell-autonomous responses that depend on phototropins and guard cell chloroplasts, and the decreased CO_2_ concentration around guard cells by mesophyll photosynthesis will cause stomatal opening [22], [23], [33], [36], [37], [38]. In other words, guard cell chloroplasts indirectly monitor photosynthetic CO_2_ fixation in mesophyll chloroplasts by absorbing PAR in the epidermis.
